# In vitro evaluation of glass fiber post

**DOI:** 10.4317/jced.50737

**Published:** 2012-10-01

**Authors:** Jasjit Kaur, Navneet Sharma, Harpal Singh

**Affiliations:** 1 M.D.S, Assistant professor. Department of Prosthodontics, Himachal Dental College, Sunder Nagar, H.P, India.; 2 M.D.S, Reader. Department of Oral Medicine and Radiology, Himachal Dental College, Sunder Nagar, H.P, India.; 3 M.D.S, Prof and head. Department of Prosthodontics, Desh Bhagat Institute of Dental Sciences, Muktsar, Punjab, India.

## Abstract

Statement of problem: Techniques and recommendations for the restoration of endodontically treated teeth have changed from the use of custom cast metal post and core system to glass fiber-reinforced (GFRC) post and composite core system. Has this latest prefabricated glass fiber reinforced post and composite core system increased the fracture resistance of teeth and reduced the incidence of unrestorable root fractures. Purpose: The purpose of this study was to evaluate the incidence of root fracture and mode of failure of endodontically treated teeth restored with two different post and core systems. Material and Methods: Forty maxillary central incisors were randomly divided into two groups. (n=20). All teeth received endodontic treatment. First group was restored with custom cast post and core system. Second group was restored with glass fiber post and composite core system. In Both the groups posts were cemented with adhesive resin cement. Compressive load was applied at an angle of 130 to the long axis of teeth at a cross head speed of 1 mm/min until fracture occurred. Data were analyzed with student “t” test P<.001. Results: The mean value for fracture resistance was (331.4025) N in Group -I Custom cast Ni-Cr post and core and (237.0625) N in Group -II Glass fiber reinforced post and composite core system. Students “t” test shows the significant difference in fracture resistance of two groups. Conclusion: This study showed that the incidence of root fracture was significantly higher in custom cast Ni-Cr post and core system than glass fiber post and composite core system. A more favourable mode of failure was observed in teeth restored with Group II glass fiber post system.

** Key words:**Post-and-core technique, glass fiber post, cast post and-core system, fracture resistance, endodontically treated teeth.

## Introduction

The restoration of endodontically treated teeth has been studied extensively (1). Posts are widely used for the restoration of the teeth when there is insufficient coronal tooth structure to retain a core for the definitive restoration (2). Cast posts and cores are commonly advocated for teeth with little remaining coronal structure or for uniradicular teeth with small coronal volume (3).

Endodontically treated anterior teeth have traditionally been restored with cast or wrought metal posts and cores. These metallic posts have a much higher modulus of elasticity than the supporting dentine; this mismatch in modulus could lead to stress concentration and leads to failure. This has lead to search for a plastic based material that has modulus closer to that of dentine (4).

Tooth-colour posts have increased in popularity since they were introduced in 1997 (5). Prefabricated post sys-tems have become more popular because they can provide satisfactory results while saving chair time and reducing costs (6). Tooth-colour fibe-reinforced posts have esthetic advantages, including increased transmission of light through the root and the overlying gingival tissues. Moreover, fiber-reinforced posts eliminate the problems of corrosive reactions that can occur with metal alloy prefabricated posts. Fiber- reinforced posts also have the advantage of easy removal if endodontic retreatment is required. An important characteristic of fiber-reinforced posts is their elastic modulus, which is similar to that of dentin, resin cements, and resin core materials (7).

The cement used for cementation with glass fiber post is resin adhesive cement. This cement provides stronger union between post and core and tooth structure using adhesive bonding technique. This adhesive restorative cement transmits and distributes functional stresses across the bonding interface to the tooth more properly (8). Integration of adhesive technique into post and core procedures resulted in “monobloc” type of restoration (9). So in glass fiber post and composite core system along with resin adhesive cement resulting in “monobloc” type of restoration which transmits and distributes functional stresses across the tooth more properly. On the basis of these in vitro or in vivo studies (10) fiber post composite restorations have been recommended because they improve teeth flexibility under applied loads as well as stress distribution between post and dentin (11), to reduce the risk of root fracture, the most serious type of failure (12).

The purpose of this study was to evaluate the incidence of root fracture and mode of failure of endodontically treated teeth restored with two different types of posts custom cast Ni-Cr post and core system; glass fiber reinforced post and composite core system.

## Material and Methods

Forty, freshly extracted human maxillary central incisors were selected for the study. The teeth were collected irrespective of the age, sex, and side of the arch. All the teeth were without root caries, root fillings, root cracks, and minimum of 10mm of root length. The selected teeth were stored in artificial saliva (wet mouth ICPA C09002) at room temperature until used for study, to avoid their dehydration. All the teeth were decoronated horizontally at cemento-enamel junction, perpendicular to long axis of teeth. Endodontic treatment of selected teeth was completed as: A#15-50 using step back technique. After endodntic treatment all the prepared teeth were randomly divided into two groups of twenty teeth each,

Group I- In this group twenty samples of endodontically treated teeth is restored with Custom cast Ni-Cr alloy post and core (CP) system. (Wiron-99, Bego, USA)

Group II- In this group twenty samples of endodontically treated teeth is restored with Glass fiber-reinforced post and composite core (GFRP) system. (Glassix, Nordin, Switzerland)

Group I (CP) Peeso reamers (#1-6, Mani Japan) were used to prepare the post space. For Group II (GFRP), special drills supplied with the kit, were used to prepare post space, leaving 3 mm apical seal in the root canal. Prepared post space was evaluated with the radiograph.

For Group I (CP) - Wax pattern for cast post and core was prepared. Paper pin was roughened and dipped in molten blue inlay wax and inserted into the canal. Incremental addition of wax was done to make a post pattern. The core was built up to achieve a desired core height of 4 mm, and bucco-lingual and mesio-distal dimensions 1.5 mm less than to the corresponding total bucco-lingual and mesio-distal dimensions of the specimen. The post and core wax pattern was sprued and invested. Casting was done with Ni-Cr alloy (Wiron-99, Bego, USA) and casting of post and core were obtained and finished.

For Group II (GFRP), the prefabricated glass fiber posts were cemented with adhesive resin cement (3M) ESPE (N120174). Then it was light cured for 40 sec to achieve complete polymerization. Core build up was done with Tetric N-cerem, Ivoclar Vivadent (N-12096). The preformed polyester matrix was filled with the core build up material placed on the specimen. Each increment was light cured for 60 sec. It was finished to the final core height of 6 mm and bucco-lingual and mesio-distal dimensions corresponding to that of the tooth, with the help of composite finishing kit.

Cementation of cast post in Group I (CP) were done with adhesive resin cement. The procedure of cementation was same as done for group II (GFRP), post system.

With free hand all preparations were finished with a diamond bur (DIA BURS, WR-13 ISO 068/042) at high speed with water spray (W&H Dentalwerk Burmose Gmbh Austria). All finish lines were placed at level of cemento-enamel junction. Single coat of spacer was applied to the core part of the specimen. The core was dipped into the molten blue inlay wax, to give a uniform layer of wax over the entire surface. Crown patterns were prepared for two groups of post and core system by duplicating polycarbonate crown forms (size 10).Investing and casting was done with the Ni-Cr alloy(Wiron-99, Bego, USA) . Castings were retrieved, finished and polished.

**Figure 1:**
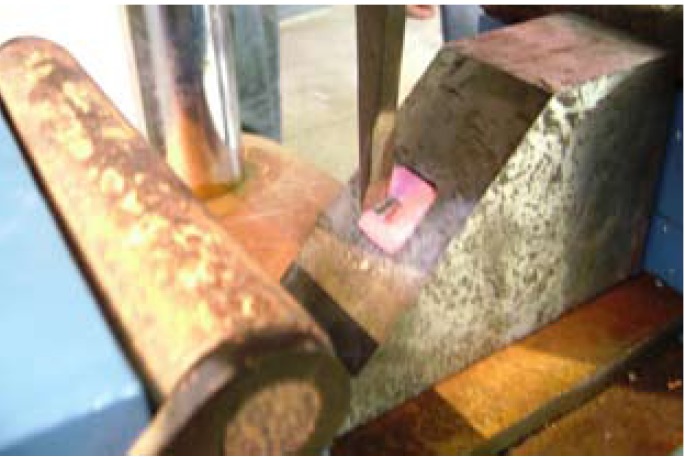
Testing specimen on universal testing machine.

**Figure 2:**
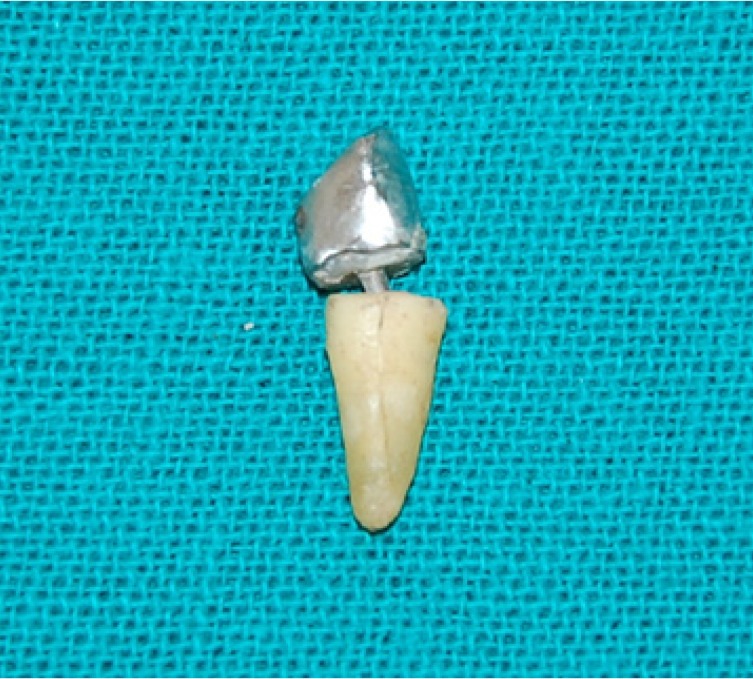
Mode of Failure in Cast Post and Core System.

**Figure 3:**
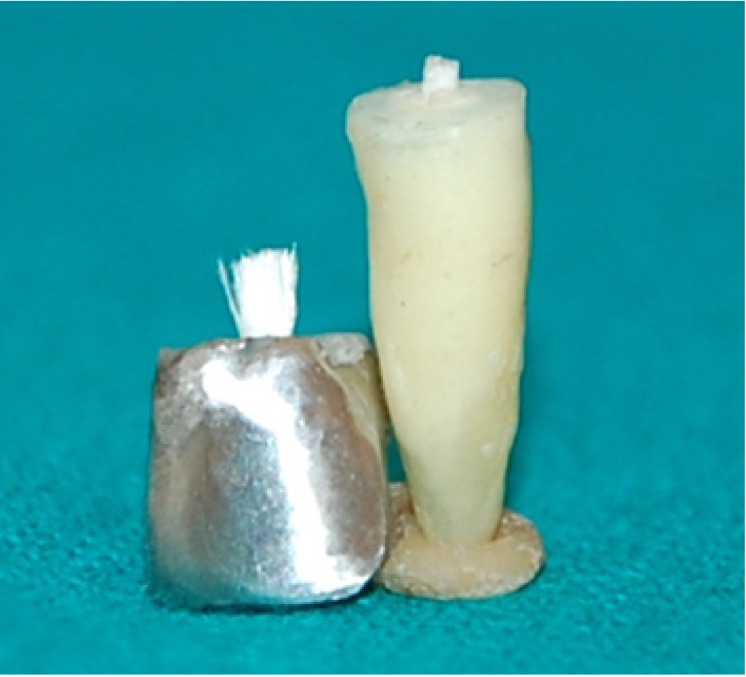
Mode of Failure in Glass Fiber Post and Composite Core System.

Cementation of crown was done with the adhesive resin cement. All specimens roots were dipped into molten wax to a depth of 2mm below the CEJ to give a uniform layer 0.2-0.3 mm of wax over the entire root surface.40 Acrylic resins blocks were fabricated by mixing the self- cure acrylic resin DPI-RR cold cure P-13103, L-13102 and pouring it into block – holder part of metal jig. Specimens were held perpendicular into the center of block– holder part of metal jig. After setting of acrylic resin, dewaxing was done to achieve a space for simulated periodontal ligament. Type I- regular viscosity regular body, polyvinyl siloxane impression material (Reprosil-Dentsply) 100313 was applied over the root surfaces and also into the mold surface in acrylic resin blocks. Each specimen was reinserted into acrylic blocks up to 2mm below CEJ and held under digital pressure until material was set. Excess material was removed with the help of B. P. knife. All specimens were prepared and stored in artificial saliva until tested. Stainless steel metal attachment tool were custom made of the desired dimensions according to the testing machine. Metal jig had the provision for holding acrylic resin blocks that orient the specimen at an angle of 130 degree to the load application tip of the attachment tool. The whole as-sembly was fitted in the universal testing machine (UTK10, Krystal industries, Maharashtra, India) and load was applied on the palatal surface of cast crown, 2mm from incisal edge, at an angle of 130 degrees to the long axis of the root, at a cross head speed of 1mm\min until failure occurred as seen in (Fig. 1). The mean load at the time of failure in custom cast post and core group is (331.4025) N (as shown in  Table 1). The mean load at the time of failure in glass fiber reinforced post and composite core was (237.0625) N (as shown in  Table 2). The time during the failure was not taken ito consideration. Each specimen was then removed from acrylic resin block, polyvinyl siloxane coating was removed and mode of failure was recorded (Fig. 2, Fig. 3).

**Table 1:**
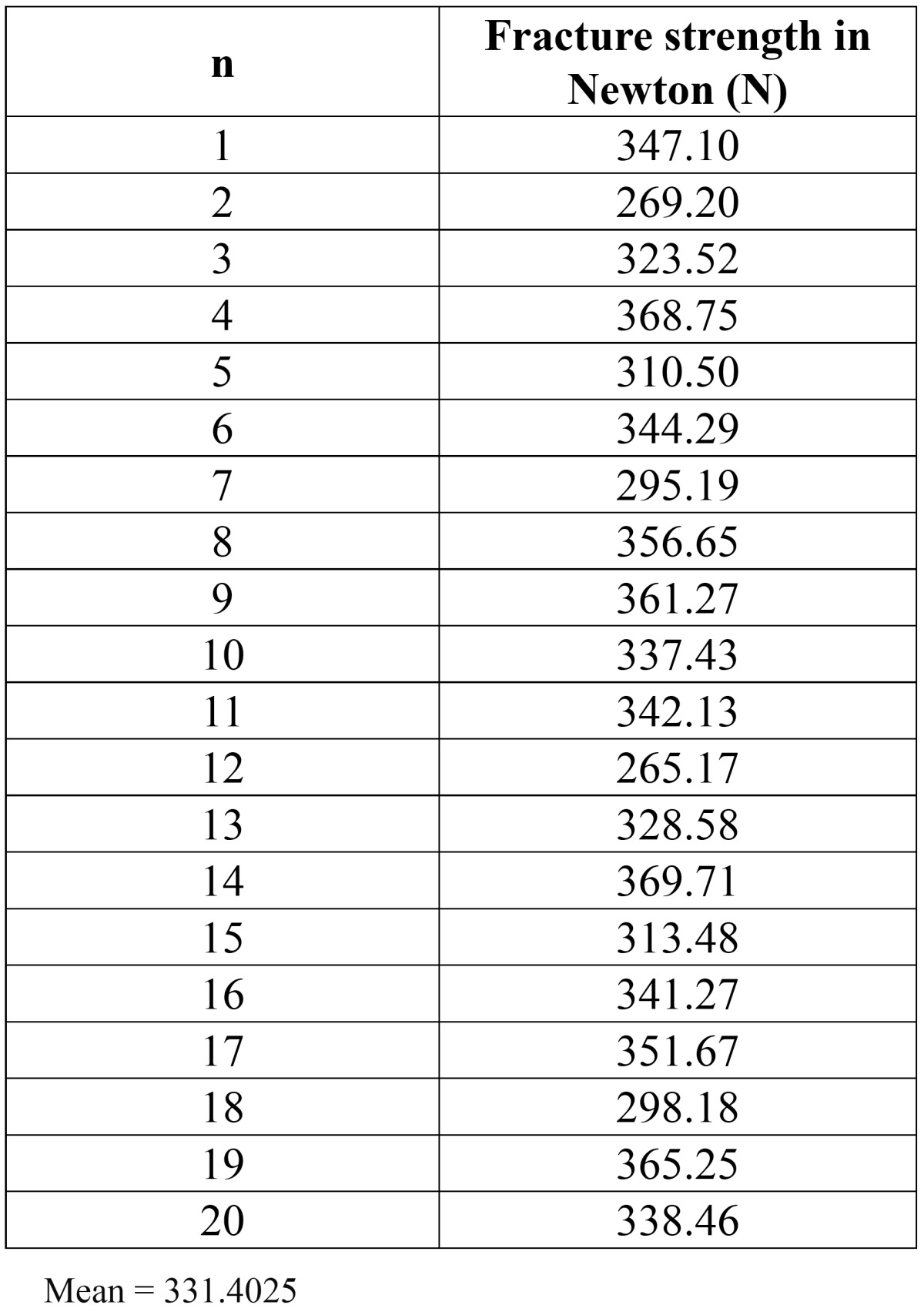
Fracture strength for Group I. Custom cast Ni-Cr post and core (n=20).

**Table 2:**
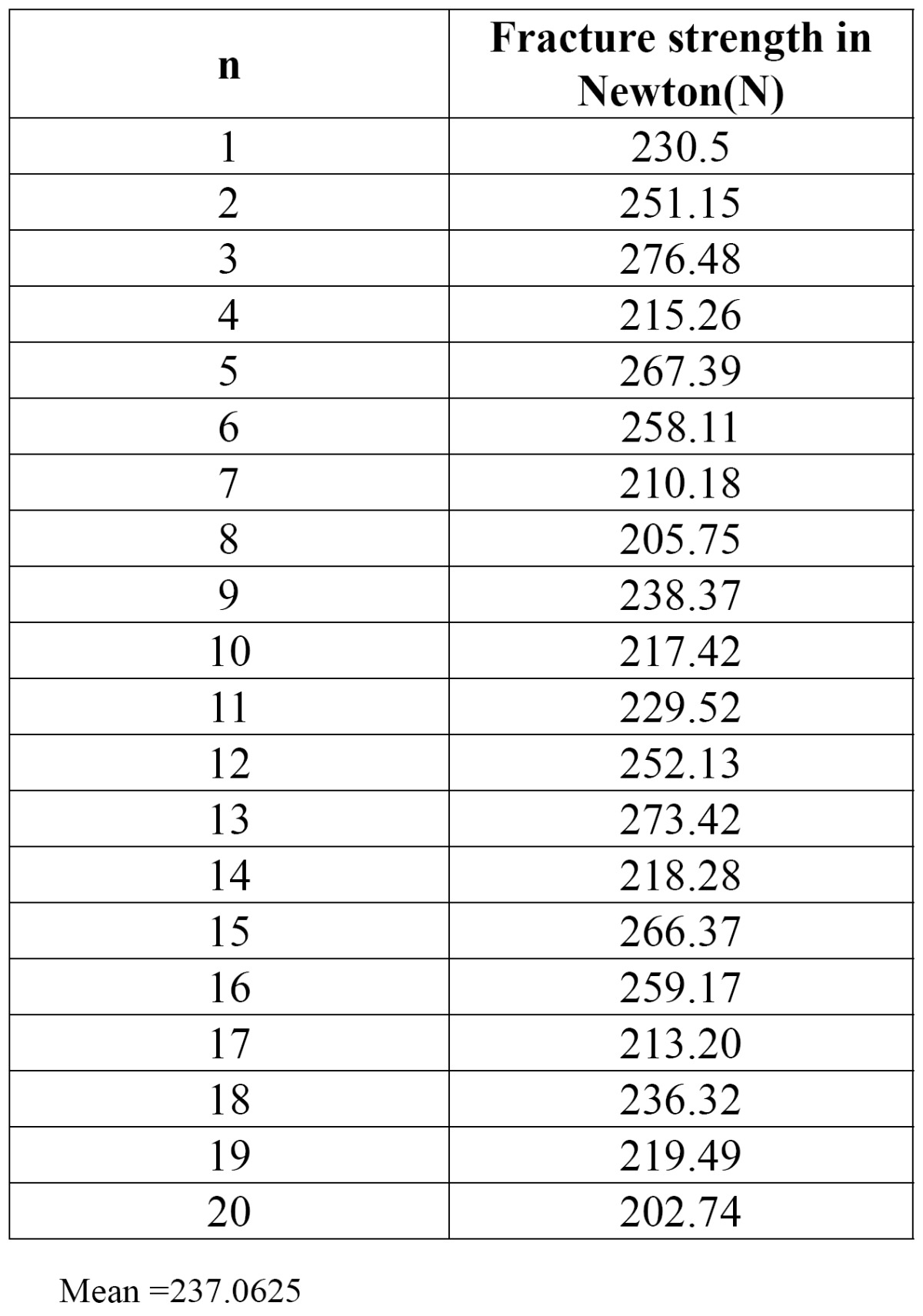
Fracture strength for Group II. Glass fiber reinforced composite post and core (n=20).

## Results

**Figure 4:**
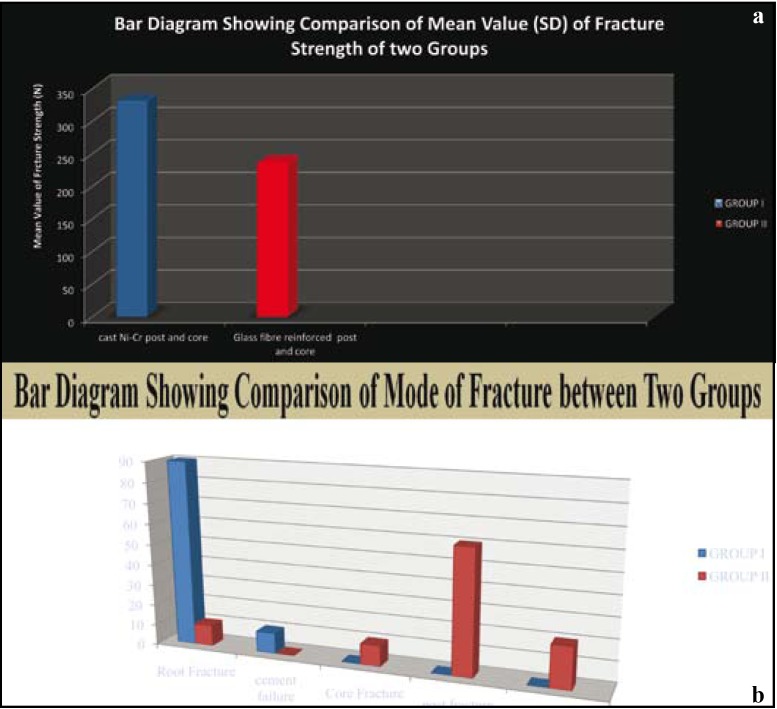
a- Comparison of mean value (SD) of fracture strength of two groups.b- Comparison of mode of fracture between two groups.

The mean fracture resistance of Custom Cast Ni-Cr post and core group and Glass fiber reinforced composite post and core group was (331.4025) N, (237.0625) N respectively. (As shown in Fig. 4). Standard deviation for group I (CP) & group II (GFRP) is 30.90574 and 24.15638 respectively (as shown in  Table 3). As standard deviation for group II (GFRP) is less than the group I (CP) so group II shows the more promising and consistent results as compared to group I (CP).

**Table 3:**
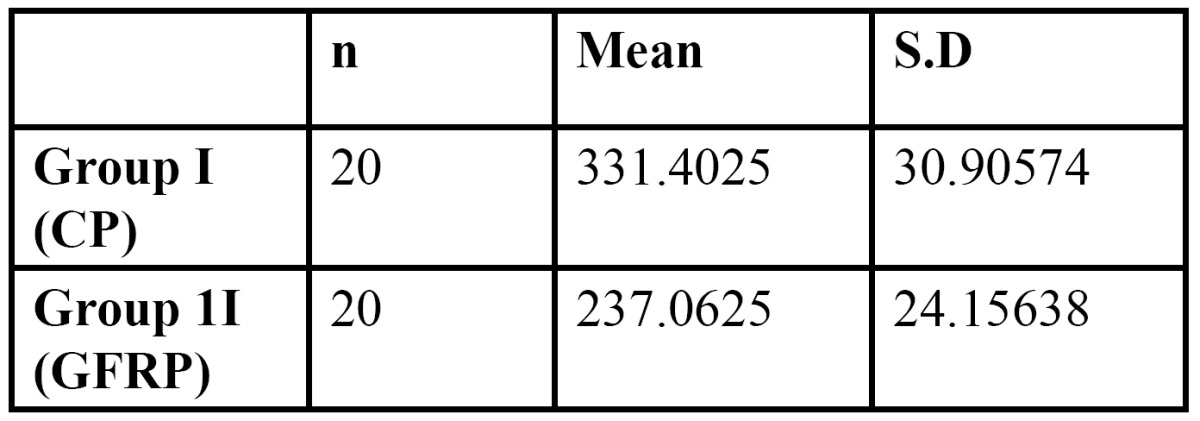
Comparison of Mean and Standard Deviation of two groups.

Mode of failure in group I Custom cast Ni-Cr post and core (CP) system was as follow- 90% of the specimens had root fracture, 10% of the specimens had cement failure. Post fracture, core fracture and post-core junction fractures were not observed in this group (as shown in Fig. 4). In group II Glass fiber reinforced post and composite core (GFRP) system 10% of the specimens shown root fracture and core fractures. 60% of the specimens had post fracture. Cement failure was not observed in this group (as shown in Fig. 4).

## Discussion

The result of this investigation suggests that fracture resistance and mode of failure of two post system do not have similar behaviour under the same experimental design. Teeth restored with group II shows the more promi-sing and consistent results as compared to group I as shown from above values.

The Group-II (GFRP) had shown the more favourable mode of failure as only 10% of the specimens had shown the root fracture as compared to the Group-I (CP) in which 90% of the specimens had shown the root fracture. The cast restorations had highest modulus of elasticity (220 GPa) as compared glass fiber post (13-40 GPa) whereas the modulus of elasticity of dentine is (15-25 GPa). In glass fiber post and composite core system modulus of elasticity is similar to dentin had resulted in improved stress distribution between the post and dentin thus resulting in improved flexibility of teeth under applied loads. Fiber posts contribute to minimizes the risks of unrestorable root fractures (13,14). It has been observed that the use of intraradicular posts adhered to both dentin and coronal core provides better distribution of forces along the root canal, contributing to the tooth reinforcement (15-18).

While in case of cast post and core system due to high difference in modulus of elasticity resulting in stress concentration at the apical region of root leads to catastrophic root fractures. These findings are shown in other studies conducted by *Insua et al* (12), *Sidoli GE et al* (19), *Sirimai S et al* (20).

The cement used in this study was resin adhesive cement. Adhesive system for post cementation improves marginal adaptation with improved seal (21-23). Relieves stresses within the root (24), optimizes fracture patterns in regards to re-restoration (25-26), and increases failure resistance compared with conventional cementation (27) at least for maxillary incisors (28).

This cement provides stronger union between post and core and tooth structure using adhesive bonding technique. *Mohhamad N* (8) claimed that this adhesive restorative cement transmits and distributes functional stresses across the bonding interface to the tooth more properly. Integration of adhesive technique into post and core procedures resulted in “monobloc” type of restoration. So in glass fiber post and composite core system along with resin adhesive cement resulting in “monobloc” type of restoration which transmits and distributes functio-nal stresses across the tooth more properly. These findings are shown in other studies conducted by *Cormier CJ* (9), *Tay FR* (29).

A few Incidence of post fracture are observed in glass fiber post and core system. This can be due to the weak bond between the internal glass fibers and resin matrix (30). In case of post fracture, mode of failure is repairable. Retreatment is done by replacing with new post system but root fractures are always non repairable.

Within the limitation of this study, it was concluded that a more favourable mode of failure was observed in teeth restored with Group II glass fiber post and composite core system. Teeth restored with Group I custom cast post system showed catastrophic vertical root fractures which are non repairable. So glass fiber post and composite core system have been recommended because they improve teeth flexibility under applied loads as well as stress distribution between post and dentin to reduce the risk of root fracture, the most serious type of failure.
